# Healthcare preferences of the general Chinese population in the hierarchical medical system: A discrete choice experiment

**DOI:** 10.3389/fpubh.2022.1044550

**Published:** 2022-11-17

**Authors:** Ni Yan, Taoran Liu, Yuan Xu, Xuanbi Fang, Xinyang Ma, Meng Yang, Jianhao Du, Zijian Tan, Er-wen Fan, Jian Huang, Babatunde Akinwunmi, Casper J. P. Zhang, Wai-Kit Ming, Liangping Luo

**Affiliations:** ^1^Department of Medical Imaging Center, The First Affiliated Hospital, Jinan University, Guangzhou, China; ^2^Department of Infectious Diseases and Public Health, Jockey Club College of Veterinary Medicine and Life Science, City University of Hong Kong, Hong Kong, Hong Kong SAR, China; ^3^Department of Public Health and Preventive Medicine, School of Medicine, Jinan University, Guangzhou, China; ^4^Singapore Institute for Clinical Sciences (SICS), Agency for Science, Technology, and Research (A*STAR), Singapore, Singapore; ^5^Department of Epidemiology and Biostatistics, School of Public Health, Faculty of Medicine, Imperial College London, London, United Kingdom; ^6^Department of Obstetrics and Gynecology, Brigham and Women's Hospital Boston, Boston, MA, United States; ^7^Center for Genomic Medicine (CGM), Massachusetts General Hospital and Harvard Medical School, Harvard University, Boston, MA, United States; ^8^School of Public Health, The University of Hong Kong, Hong Kong, Hong Kong SAR, China

**Keywords:** discrete choice experiment, healthcare preferences, hierarchical medical system, health insurance, chronic non-communicable diseases, acute infectious diseases, major diseases

## Abstract

**Background:**

Chinese health insurance system faces resource distribution challenges. A patient-centric approach allows decision-makers to be keenly aware of optimized medical resource allocation.

**Objective:**

This study aims to use the discrete choice model to determine the main factors affecting the healthcare preferences of the general Chinese population and their weights in the three scenarios (chronic non-communicable diseases, acute infectious diseases, and major diseases).

**Methods:**

This study firstly identified the key factors affecting people's healthcare preferences through literature review and qualitative interviews, and then designed the DCE questionnaire. An online questionnaire produced by Lighthouse Studio (version 9.9.1) software was distributed to voluntary respondents recruited from mainland China's entire population from January 2021 to June 2021. Participants were required to answer a total of 21 questions of three scenarios in the questionnaire. The multinomial logit model and latent class model were used to analyze the collected data.

**Results:**

A total of 4,156 participants from mainland China were included in this study. The multinomial logit and latent class model analyses showed that medical insurance reimbursement is the most important attribute in all three disease scenarios. In the scenario of “non-communicable diseases,” the attributes that participants valued were, from the most to the least, medical insurance reimbursement (45.0%), hospital-level (21.6%), distance (14.4%), cost (9.7%), waiting time (8.3%), and care provider (1.0%). As for willingness to pay (WTP), participants were willing to pay 204.5 yuan, or 1,743.8 yuan, to change from private hospitals or community hospitals to tertiary hospitals, respectively.

**Conclusions:**

This study explores the healthcare preferences of Chinese residents from a new perspective, which can provide theoretical reference for the refinement of many disease medical reimbursement policies, such as developing different reimbursement ratios for various common diseases and realizing rational configuration of medical resources.

## Introduction

A patient-centric approach allows decision-makers to be keenly aware of optimized medical resource allocation. When people seek medical help, they will make a trade-off after weighing the benefits and costs. This indicates the importance of healthcare distribution, which is critical for decision-makers to map a wellrounded strategy (1, 2). Healthcare preference was defined as “statements that indicate the importance of specific aspects of clinical behavior of care providers or the organization of care (3). Governments and health technology assessment (HTA) agencies have incorporated public preferences into the decision-making process, such as the Grading of Recommendations Assessment, Development, and Evaluation (GRADE), adopted by the American College of Rheumatology (ACR) (4).

Meanwhile, several patient groups have joined committees and citizen juries (5, 6). For example, the United States (US) Food and Drug Administration (FDA) advocates for an open attitude toward patients” recommendations to develop new drugs (7). The European Medicines Agency (EMA) also launched a pilot project to recruit patients to join the Human Medicines Committee (8). In this way, authorities can make decisions that cater to public preferences and manage scientifically.

China has established the world's largest health insurance system. In the past 15 years, the nationwide coverage rate soared from 29.7 to 97.1% (9, 10). However, this system still faces resource distribution challenges. The Chinese government has vigorously advocated a hierarchical medical system in recent years. Diseases are graded according to the severity, urgency, and therapeutic difficulty, and different medical institutions receive patients of varying severity accordingly. However, China continues to be stuck in two dilemmas: the first is the irrational distribution of medical resources, and the second is the scarcity of medical resources. The “Hospital Hierarchical Management Standard” divides China's public hospitals into three categories in contrast to other nations, based on evaluation parameters such as (1) hospital scale, (2) scientific research specialization, (3) talent strength, and (4) medical facilities. High-level hospitals, such as university-affiliated hospitals distributed unequally in different parts or cities of China, which result in that larger cities occupying a larger proportion of medical resources. And these factors have led to only a little progress in the hierarchical medical system that has been achieved (11). Additionally, even if some of the patients have received comprehensive treatment in ordinary hospitals, they are still more inclined to seek medical treatment in high-level hospitals (12), regardless of longer waiting time, which leads to congestion in high-level medical institutions and waste of medical resources, and some patients who should have been given priority for treatment missed the best time for treatment. The government realized that improving facilities alone was no longer enough to cope with the current situation. At the same time, multi-faceted resolutions may make significant progress (13), and the discrete choice experiment (DCE) is one of the methods that can quantify the public's healthcare preferences (14).

In previous studies, preference was typically measured by asking participants to directly assess each attribute related to decision-making. For example, the retrospective study of Yu- et al. (15) in China, found patients' preferences vary in the scenarios of mild, chronic and serious illness; specifically, people prefer community health facilities health in the scenario of mild illness, while general hospitals were preferred in the scenario of chronic and serious illness. The study of Wan et al. (16) found that people's preferences toward primary healthcare closely correlated with their socioeconomic status and health status. Yu et al. (17) investigated healthcare services preference among hypertension patients in China found that hypertension patients prefer treatment effect and travel time to healthcare facilities the most. Therefore, there is an evident research gap exists between the current study and previous studies in China, i.e., (1) The existing literature mainly focuses on a specific disease, without concerning different situations, e.g., focusing on the specific disease such as hypertension, without concerning infectious diseases, especially during the COVID-19 epidemic outbreak (2) Most of the existing studies only applied a single model, e.g., only using univariate analysis or logistic regressions; and (3) The key factor of “medical insurance reimbursement ratio” has been attached less importance in the existing research, since most previous studies focus on other attributes such as waiting time, care provider, etc.

At the same time, DCE can be used for joint analysis through the selection process between two or more treatment options (each is a combination of different attributes) (18), which can apply to the qualitative measurement for various interventions, products, or policies. Nowadays, DCE is also extensively adopted in the medical field to assess healthcare priorities, mainly for selecting therapeutic drugs and regimens.

The published studies on DCE mainly focus on evaluating the efficacy, safety, and medical convenience of diseases (mainly metabolic diseases such as diabetes) in European and American countries (19, 20). Few studies were performed on healthcare preferences in China (21, 22). The main objective of this study to investigate the healthcare preferences of the general population in China and discuss how the unique medical insurance system affects people's choice of hospitals, and find the latent factors that have a large impact on peoples' trade-offs when they seek medical services. These results may help decision-makers determine the priority of medical resource allocation and adjust medical policies to provide more efficient medical services for the public and provide insights for policymakers to implement appropriate policies to help divert patients from high-level hospitals to primary hospitals, and then help avoid the waste of medical resources, and improve the current practice of the hierarchical medical system.

## Methods

### Study design and procedure

DCE is based on the random utility theory developed by Thurstone (23), which quantifies the importance of various attributes by stimulating participants' preferences. According to this theory, products are filed under different attributes, and each attribute is classified into different levels. Participants stimulate preferences by repeatedly selecting among combinations of these attributes and levels. Thus, the relative importance of each attribute is determined.

We obtained the initial attributes and levels through an extensive literature search, among which the most commonly used attributes included waiting time, quality of medical services, type of doctors, and cost (24–26). To further screen the crucial attributes, semi-structured interviews were conducted with 21 participants (ages ranging from 21 to 69), who were asked to list and rank them by importance. The final attributes in the questionnaire were determined after discussion with two experts from the fields of health economics and public health, and the phrasing in the questionnaire was revised based on the feedback of 21 interviewed participants. All attributes and levels in the questionnaire are shown in Table 1. Notably, medical insurance reimbursement occupies a relatively important position in most participants' consideration, which previous surveys have never included. Considering the peculiarity of the Chinese medical insurance system, we decided to add this attribute in the questionnaire after expert discussion. Levels of attributes were set after a literature review and using data from the China Health Statistics Yearbook 2020 (9), covering the involved maximum and minimum values. Unlike other countries, three levels of institutions comprise China's healthcare system: community hospitals (responsible for primary healthcare in communities and counties), secondary hospitals, and tertiary hospitals (providing municipal, provincial and national medical help).

**Table 1:** Attributes and levels.

**Attributes**	**Levels**	**Definition**
Hospital level	Community hospital Secondary hospital Tertiary hospital Private hospital	Public hospitals in China are divided into three grades according to their functions, facilities and technical strength, among which tertiary hospitals are the highest.
Distance	Within the county/district Within the cityv Within the province Across the province	The location of clinics.
Care provider	Resident Attending physician Associate chief physician Consultant/chief physician	According to the top-down order, the professional titles of Chinese doctors are from low to high.
Waiting time	No need to wait 3 days 6 days 9 days 12 days	Includes appointment registration time and hospital waiting time.
Cost (CNY)		
Chronic non-infectious diseases(e.g., diabetes)	300 600 900 1,200	Treatment cost per patient per time (including registration fee, drug fee, hospitalization fee, etc.)
Acute infectious diseases (e.g., COVID−19)	5,000 10,000 15,000 20,000	
Major diseases (e.g., lung cancer)	20,000 40,000 60,000 80,000	
Medical insurance reimbursement	0 20% 40% 60% 80% 100%	Expenditure from the Medical Insurance Fund.

### DCE instruments

Theoretically, the scenario combination should be, which is unreasonable to operate. In this study, Lighthouse Studio (version 9.9.1) was used to create the fractional factorial design method—based on two principles (27): (1) orthogonality and (2) balance—to help determine the maximum number of scenarios, which ensures no correlation among levels and attributes and equal probability to appear in each set of task choices in under 3 types of disease assumption. Six random scenarios and one fixed scenario for quality control were set in all scenarios.

### Questionnaire

Our questionnaire was divided into two parts. The first section collected demographic information, including age, sex, educational background, income level, nationality, household registration, and medical insurance types. Noticeably, household registration in China includes two kinds: rural and urban (28). Although the mobility in-between is increasing, the population gap remains. Studies have shown that residents with rural household registration have a lower social status and inadequate access to healthcare services (29). The second part consists of chronic non-communicable diseases, acute communicable diseases, and major diseases. In chronic non-communicable diseases, we take diabetes as an example to explore participants' preference for medical services because diabetes is responsible for a higher prevalence of chronic diseases in China than other diseases. Its cost is centrally allocated to secondary and tertiary hospitals than primary institutions (9). Therefore, the preferences in diabetes can serve as a perfect instance in our study and provide a prototype discernment. In the context of acute infectious diseases, we chose COVID-19 as an example because of its global impacts since early 2020. We anticipate that this approach can deepen participants' understanding and provide a decision-making basis to deal with future outbreaks of acute diseases (30). In major disease situations, lung cancer serves as an example because lung cancer is the most prevalent cancer in China, with an age-standardized incidence rate (ASIR) of 35.92/10^5^ (31). Lung cancer is also the leading cause of cancer death in most parts of the world. Each situation includes seven questions to describe the assumption that, given the hypothetical situation, participants should repeatedly make choices among medical schemes with the same format but different combinations of attributes and levels (Table 2 shows an example of the selecting interface). A detailed explanation of the purpose and definition of each attribute was provided to ensure the comprehensibility of the questionnaire. Each question included three options: option A, option B, and “Neither,” and participants were entitled to withdraw from the study at any given time. This method can obtain the influence of different attributes on patient selection.

**Table 2:** Example of the selecting interface.

	**Option A**	**Option B**	**Neither**
Hospital level	Community hospital	Tertiary hospital	
Distance	Within the county/district	Within the city	
Care provider	Associate chief physician	Consultant/ Chief physician	
Waiting time	No need to wait	3 days	
Cost (CNY)	600	900	
Medical insurance reimbursement	80%	60%	
	Select	Select	Select

A rationality test was performed with a fixed option in each scenario to ensure the quality of the questionnaire, which included the best choice and the worst choice (e.g., the nearest, cheapest and best-equipped hospitals vs. the farthest, most expensive and standard-equipped hospitals). If participants chose the worst one, they were considered to have failed the rationality test. To maintain the quality and feasibility of the questionnaire, 238 volunteers were recruited to do the pilot, and slight adjustments were made to the controversial part of the questionnaire.

### Sampling and data collection

Our anonymous self-administrated online questionnaire, produced by Lighthouse Studio version 9.9.1 (Sawtooth Software, Inc; Provo, Utah, US) software, was distributed to voluntary respondents throughout several social media apps using the snowball sampling method (32), i.e., using WeChat and Weibo as main sampling approaches. The target recruiting respondents were residents from mainland China, and the recruiting time period was from January 2021 to June 2021. Before the respondents filling the questionnaire, an online page including a consent form was presented. Respondents were required to answer if they agreed to participate in this study given the background information of the study. Respondents that failed to answer in the consent form were considered as disqualified.

### Ethical approval

The study was approved by the Medical Ethics Committee of Jinan University, and the ethical code is JNUKY-2021-004.

### Statistical analysis

#### Descriptive statistics

Descriptive statistics were applied by Stata (Version 16) to summarize the detailed number and proportion of respondents of the specific level of demographic variables. The MNL and LC models were performed using Lighthouse Studio (Version 9.9.1).

#### Multinomial logit model (MNL)

The multinomial logit model was used in this study to quantify the weights of each attribute and the utility of detailed levels in respondents' preferences. Specifically, the MNL model was established based on the principle of random utility (27), and the utility formula in this study was given by:


$$U_{n}=V_{n}+\varepsilon_{n}=\alpha_{1}+\beta_{1}X_{1n}+\beta_{2}X_{2n}+\ldots +\beta_{m}X_{mni}+\varepsilon_{n}$$

Random utility of each attribute level brought to individuals is represented by coefficient β, and ε means the fixed utility in the MNL model. Odds ratios and 95% CI of levels according to the reference level in each attribute were calculated better visualize the rise or the decline of the utility change. A sub-group analysis using the MNL model was also conducted according to the different types of medical insurance held by the respondents. Specifically, there are four different types of medical insurance (1) Urban and Rural Resident Basic Medical Insurance (URRBMI); (2) Urban Employee Basic Medical Insurance (UEBMI); (3) Other insurance types and (4) No insurance. We aim to compare the heterogeneity among respondents' preferences under the condition of different medical insurance types.

#### Latent class model (LC)

The MNL model's inherent properties make it the most advantageous model for this study's topic and data type. However, the limitation of the MNL model is also evident. The MNL can only provide the statistical analysis for the whole sampled population, which means it cannot tell the preference heterogeneities of different groups of respondents among the whole sampled population. Therefore, we applied the LC model simultaneously with the MNL model. LC model is an effective tool to help identify heterogeneities of preference of several latent subgroups of individuals among the whole respondent population (29). For example, the formula of the LC model (30) for an observed item response pattern y out of an array of response patterns Y is defined as below:


$$P\left(Y=y\right)=\overset{C}{\underset{c=1}{\sum }}P\left(L=c\right)\overset{J}{\underset{j=1}{\prod }}P\left(Y_{j}=y_{j}|L=c\right)$$

The C is the number of the latent subgroups, P(L=c) represents the unconditional probabilities that should sum to 1 and represents the conditional probabilities. The process of determining the number of subgroups of the LC model is shown in detail in Appendix 1 (33–36).

#### Willingness to pay (WTP)

Willingness to pay (WTP) of attribute levels was calculated based on the continuous attribute cost (in our study, the diagnosis expense). WTP is an economic concept that measures the optimal price that an individual is willing to pay for the product. In our study, the WTP provides a relatively intuitive measure of how much the participants are willing to sacrifice from one attribute level to another. It also visually provides policymakers with suggestions about the improvement points in future policymaking.

## Result

### Descriptive Statistics

A total of 4,156 participants who consented to the study met the criteria and completed the questionnaire, of which 357 participants were excluded because of unreasonable completion time or failure to complete the rationality test. Finally, we analyzed the data of 3,841 participants. The characteristics of the respondents are shown in Table 3. Both sexes were equally represented; 49.8% were male, 49.9% were female, and age was mainly concentrated in the 18–25 (38.7%) and 26–35 (29.0%) groups.

**Table 3:** Characteristics of the study sample.

**Characteristics**	**Full sample *n* = 4,156**	**Analysis sample: *n* = 3,841** **(Who passed the rationality test)**	**Excluded sample: *n* = 315 (Who failed the rationality test)**
	***n* (%)**	***n* (%)**	***n* (%)**
**Sex**	
Male	2,098 (50.48)	1,913 (49.80)	185 (58.73)
Female	2,046 (49.23)	1,916 (49.88)	130 (41.27)
Other	12 (0.29)	12 (0.31)	0 (0.00)
**Age**	
Under 18	157 (3.78)	145 (3.78)	12 (3.81)
18–25	1,593 (38.33)	1,488 (38.74)	105 (33.33)
26–35	1,212 (29.16)	1,114 (29.00)	98 (31.11)
36–45	684 (16.46)	619 (16.12)	65 (20.63)
46–55	307 (7.39)	277 (7.21)	30 (9.52)
56–60	110 (2.65)	106 (2.76)	4 (1.27)
Above 60	93 (2.24)	92 (2.40)	1 (0.32)
**Educational background**	
Middle school education or below	364 (8.8)	347 (9.03)	17 (5.40)
High School education	596 (14.34)	545 (14.19)	51 (16.19)
Vocational school education	885 (21.29)	803 (20.91)	82 (26.03)
Bachelor's degree	2,017 (48.53)	1,876 (48.84)	141 (44.76)
Master's degree	263 (6.33)	245 (6.38)	18 (5.71)
PhD degree	31 (0.75)	25 (0.65)	6 (1.90)
**Occupation**	
Students	668 (16.07)	645 (16.79)	23 (7.30)
Head of state organs, party organizations, enterprises	528 (12.70)	492 (12.81)	36 (11.43)
Professional and technical personnel	880 (21.17)	805 (20.96)	75 (23.81)
Officers and related personnel	637 (15.33)	569 (14.81)	68 (21.59)
Business, service personnel	851 (20.48)	794 (20.67)	57 (18.10)
Agricultural, forestry, animal husbandry	309 (7.44)	276 (7.19)	33 (10.48)
Production and transportation equipment operators	228 (5.49) 14	210 (5.47)	18 (5.71)
Military	(0.34)	14 (0.36)	0 (0.00)
Others	41 (0.99)	36 (0.94)	5 (1.59)
**Registered residence**	
Rural	2,526 (60.78)	2,341 (60.95)	185 (58.73)
Urban	1,630 (39.22)	1,500 (39.05)	130 (41.27)
**Monthly income (CNY)**			
Under 5,000	1,831 (44.06)	1,739 (45.27)	92 (29.21)
5,000–10,000	1,756 (42.25)	1,594 (41.50)	162 (51.43)
10,001–20,000	478 (11.50)	428 (11.14)	50 (15.87)
More than 20,000	82 (1.97)	71 (1.85)	11 (3.49)
**Insurance type**	
URRBMI	2,499 (60.13)	2,305 (60.01)	194 (61.59)
UEBMI	1,479 (35.59)	1,382 (35.98)	97 (30.79)
Other commercial insurance	124 (2.98)	104 (2.71)	20 (6.35)
No insurance	54 (1.30)	50 (1.30)	4 (1.27)

A majority of the participants (60.5%) responded that the most frequently visited medical facilities were general public hospitals (including tertiary and secondary hospitals), followed by community hospitals (18.4%) and specialized public hospitals (14.5%). In comparison, only 6.1% of respondents chose private medical institutions. When selecting hospitals, respondents attached the most significant weights to the quality of medical service (72.1%), the reputation of the hospital among peers (46.1%) and the expenses (41.1%). In this case, public hospitals represented by tertiary hospitals have absolute advantages, while community hospitals are less likely to be the first choice for most people, despite their convenience. Although community hospitals are not preferable, 84.7% of the respondents expressed willingness when asked if they would like to see a doctor in a community hospital. However, only 57.5% of respondents were willing to be hospitalized, and only 35.2% said they would agree to undergo surgery in a community hospital. Most of the respondents believed that compared to tertiary hospitals, community hospitals had the advantages of better accessibility (66.3%), shorter waiting times (64.5%), and higher reimbursement rates (53.0%). When asked why they chose tertiary hospitals over community hospitals, most expressed unwillingness that was ascribed to the outdated medical equipment, uncomfortable environment, and the low quality of medical service in community hospitals, while some respondents (18.3%) said it was the poor service attitudes of medical staff that led to fewer visits. In terms of the general acknowledgment of medical insurance, most people are informed of medical insurance through community publicity and social media such as news reports, networks, and new media communication. However, only 12.3% of the respondents were familiar with the hierarchical diagnosis and treatment system. In comparison, most of the respondents (37.8%) said they had a general understanding, and, significantly, 16.6% of the respondents had not heard of this system.

### Multinomial logit model (MNL)

#### Preference analysis

##### Chronic non-infectious diseases

**Figure 1:**
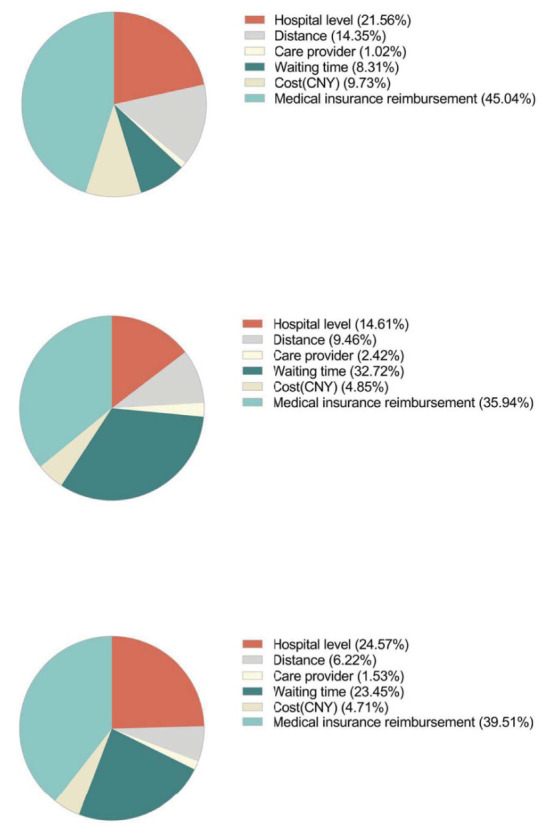
Attribute importance (MNL).

Table 4 shows the results of all attributes and levels when respondents suffered from chronic non-communicable diseases. The most important attribute for choice of hospitals is “medical insurance reimbursement” (45.0%), followed by “hospital level” (21.6%) and “distance” (14.4%) (Figure 1). Respondents appeared less concerned about “care provider,” with only 1.0% choosing that option. We used the first level of each attribute as a reference to calculate the odds ratio of each level. If the odds ratio was >1, people were more likely to choose this level than the reference level. For example, with “tertiary hospital” as the reference grade, the OR of “secondary hospital” was 0.849 [95% CI (0.824–0.875)], indicating that respondents were more likely to choose “tertiary hospital.” Similarly, with the first level of each attribute as a reference, respondents were more likely to choose the level of “3 days,” “CNY600,” “20,” “40,” “60,” “80,” and “100%” in other attributes.

**Table 4:** Attributes and levels of chronic non-infectious diseases (MNL).

**Attributes levels**	**Coefficient**	**Standard error**	**OR (95%CI)**	WTP (CNY)^*^
**Hospital level**	
Tertiary hospital	0.307***	0.016	Reference	Reference
Secondary hospital	0.143***	0.015	0.849 (0.824–0.875)	585.0
Community hospital	−0.182***	0.016	0.613 (0.595–0.633)	1,743.8
Private hospital	−0.267***	0.016	0.564 (0.547–0.581)	2,045.0
**Distance**	
Within the county/district	0.144***	0.016	Reference	Reference
Within the city	0.081***	0.016	0.938 (0.910–0.968)	-287.8
Within the province	0.012	0.015	0.876 (0.850–0.903)	470.5
Across the province	−0.237***	0.016	0.683 (0.662–0.704)	1,361.0
**Care provider**	
Resident	0.014	0.015	Reference	Reference
Attending physician	0.001	0.016	0.987 (0.957–1.018)	46.5
Associate chief physician	−0.013	0.016	0.973 (0.944–1.004)	96.4
Consultant/chief physician	−0.001	0.016	0.985 (0.955–1.016)	53.6
**Waiting time**				
No need to wait	0.097***	0.018	Reference	
3 days	0.099***	0.018	1.003 (0.967–1.040)	64.8
6 days	−0.035**	0.018	0.877 (0.846–0.909)	
9 days	−0.039**	0.018	0.873 (0.842–0.905)	
12 days	−0.122***	0.018	0.804 (0.776–0.833)	
**Cost (CNY)**	
300	0.089***	0.016	Reference	
600	0.095***	0.016	1.006 (0.976–1.038)	
900	−0.020	0.016	0.897 (0.870–0.925)	
1,200	−0.164***	0.016	0.777 (0.777–0.801)	
**Medical insurance reimbursement**				
0	−0.607***	0.022	Reference	
20%	−0.401***	0.021	1.230 (1.180–1.281)	-42. 7
40%	−0.176***	0.021	1.539 (1.478–1.602)	
60%	0.166***	0.021	2.168 (2.082–2.258)	
80%	0.427***	0.021	2.814 (2.703–2.930)	
100%	0.590***	0.021	3.312 (3.177–3.452)	

* WTP represents the monetary index of the participants' valuation of an attribute, and describes the improved average maximum monetary equivalence in each level. WTP is calculated by the coefficient of ‘cost' and each other level,and the positive (+)/ negative (–) of WTP shows that the respondents are willing to pay or receive compensation for obtaining the reference level. ***p < 0.001 and **p < 0.01.

**Figure 2:**
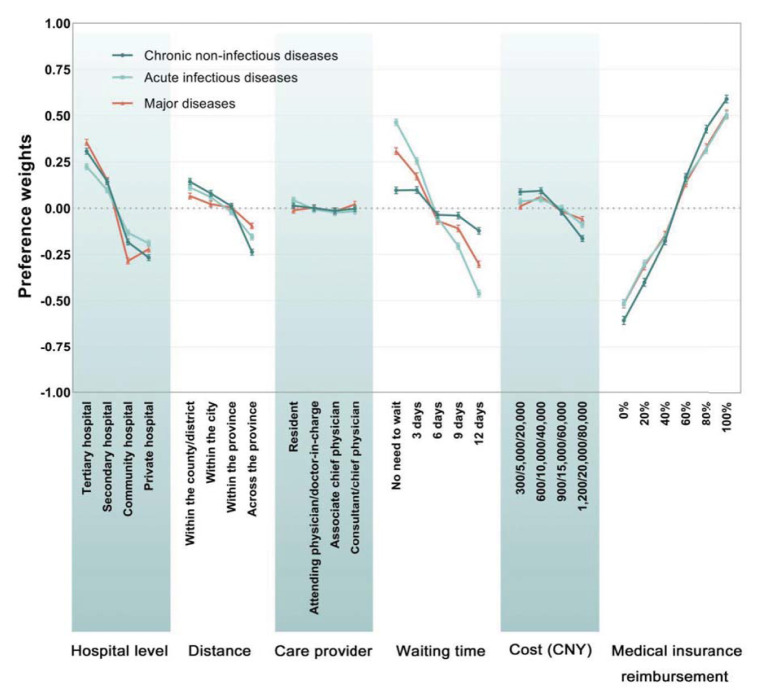
Preference weights (MNL).

To describe this more visually, the estimated preference weights of each level were drawn in Figure 2. When the coefficient is positive, it means that the level is positively correlated with the respondents' preference and vice versa. For example, at the “hospital level,” people tend to choose “tertiary hospital” and “secondary hospital.” In addition, for other attributes, respondents prefer a shorter distance, shorter waiting time, less cost and higher insurance reimbursement rates. Surprisingly, in the attribute' care provider,” people slightly prefer residents and attendings to provide medical services. The vertical distance between the preference weights of any two levels in the figure represents the utility difference. For example, utility increases 1.2 when the “health insurance reimbursement” changes from 0 to 100%. The change from “private hospital” to “tertiary hospital” will increase 0.57 in utility. However, the changes of “cost” and “care provider” did not lead to significant changes in utility. This suggests that respondents generally prefer higher medical reimbursement rates and the level of hospitals compared with changes in other attributes, and the level of doctors and the cost of medical services are not of concern.

#### Acute infectious diseases

In the acute infectious disease scenario, the most important attribute was “medical insurance reimbursement” (35.9%), followed by “waiting time” (32.7%). However, compared with chronic non-infectious diseases, the importance of “medical insurance reimbursement” in this scenario decreased by 9.1%. Noticeably, the importance of “wait time” changed greatly, increased to 33.7%, while “cost” and “care provider” are still of relatively less importance. In terms of odds ratio, compared with the reference levels, respondents are more likely to choose “CNY10,000,” “20%,” “40%,” “60%,” “80%,” and “100%.” As for preference weights (Table 5), preference in the same attribute is roughly the same as that of chronic non-infectious diseases, except for a slight difference in the “distance” attribute, in which “within the province” encounters utility loss under this circumstance. Meanwhile, the coefficient of “attending” also changed from positive to negative, indicating this kind of care provider might not be preferable.

**Table 5:** Attributes and levels of acute infectious diseases (MNL).

**Attributes levels**	**Coefficient**	**Standard error**	**OR (95%CI)**	**WTP (CNY)**
**Hospital level**	
Tertiary hospital	0.223^***^	0.016	Reference	Reference
Secondary hospital	0.099^***^	0.015	0.883 (0.857–0.910)	14,738.8
Community hospital	−0.132^***^	0.016	0.701 (0.679–0.723)	42,167.5
Private hospital	−0.190^***^	0.016	0.661 (0.642–0.682)	49,040.5
**Distance**	
Within the county/district	0.113^***^	0.016	Reference	Reference
Within the city	0.061^***^	0.016	0.950 (0.921–0.979)	−7,258.6
Within the province	−0.020	0.015	0.876 (0.850–0.903)	15,719.3
Across the province	−0.155^***^	0.016	0.765 (0.742–0.789)	31,744.9
**Care provider**	
Resident	0.045^***^	0.015	Reference	Reference
Attending physician	−0.006	0.016	0.950 (0.921–0.980)	6,086.4
Associate chief physician	−0.023	0.016	0.934 (0.906–0.963)	8,121.7
Consultant/chief physician	−0.016	0.016	0.940 (0.912–0.969)	7,310.5
**Waiting time**	
No need to wait	0.464^***^	0.018	Reference	9,149.3
3 days	0.254^***^	0.018	0.810 (0.782–0.839)	
6 days	−0.052**	0.018	0.597 (0.576–0.618)	
9 days	−0.205^***^	0.018	0.512 (0.494–0.531)	
12 days	−0.461^***^	0.019	0.396 (0.382–0.411)	
**Cost (CNY)**	
5,000	0.038^***^	0.016	Reference	
10,000	0.049^***^	0.016	1.011 (0.980–1.042)	
15,000	0.002	0.016	0.964 (0.935–0.944)	
20,000	−0.088^***^	0.016	0.881 (0.881–0.908)	
**Medical insurance reimbursement**	
0	−0.515^***^	0.022	Reference	−1,206.2
20%	−0.301^***^	0.021	1.238 (1.188–1.289)	
40%	−0.160^***^	0.021	1.425 (1.369–1.484)	
60%	0.158^***^	0.021	1.960 (1.882–2.041)	
80%	0.316^***^	0.021	2.294 (2.204–2.389)	
100%	0.502^***^	0.021	2.763 (2.652–2.879)	

*** *p* < 0.001 and **p < 0.01.

As shown in Figure 2, “medical insurance coverage” outweighs all other attributes, with the utility range of 1.02, followed by “waiting time,” in which the utility increased by 0.92. The most significant difference from the chronic non-infectious diseases scenario is that “waiting time” seems to impact more on their choice. At the same time, the importance of “medical insurance reimbursement” has diminished.

#### Major diseases

Table 6 and Figure 2 show the overall results. In the major diseases scenario, this turned out differently. The attributes “waiting time” (23.5%) and “hospital level” (24.6%) still played a relatively important role, with “medical insurance reimbursement” remaining the most influential attribute (39.5%). Compared with chronic non-infectious diseases, the importance of “waiting time” was significantly increased. Compared with acute infectious diseases, people's concern seems to move from “waiting time” to “hospital level.” In all levels, the odds ratio of “attending,” “consultant/chief physician,” “CNY40,000,” “20,” “40,” “60,” “80,” and “100%” is >1, which showed inclination compared to the reference level.

**Table 6:** Attributes and levels of major diseases (MNL).

**Attributes levels**	**Coefficient**	**Standard error**	**OR (95%CI)**	**WTP (CNY)**
Hospital level
Tertiary hospital	0.355***	0.016	Reference	Reference
Secondary hospital	0.150***	0.015	0.814 (0.790–0.839)	178,324.8
Community hospital	−0.285***	0.016	0.527 (0.511–0.544)	555,300.8
Private hospital	−0.220***	0.016	0.562 (0.546–0.580)	499,522.0
Distance
Within the county/district	0.067***	0.016	Reference	Reference
Within the city	0.023	0.016	0.957 (0.928–0.987)	-20,057.4
Within the province	0.005	0.015	0.939 (0.912–0.968)	54,333.2
Across the province	−0.095***	0.015	0.851 (0.825–0.877)	140,446.1
Care provider
Resident	−0.011	0.015	Reference	Reference
Attending physician	0.004	0.016	1.015 (0.984–1.046)	−12,70
Associate chief physician	−0.017	0.016	0.994 (0.964–1.025)	3.0 5,073.3
Consultant/chief physician	0.023	0.015	1.035 (1.004–1.066)	−29,491.1
Waiting time
No need to wait	0.308***	0.018	Reference	
3 days	0.172***	0.018	0.873 (0.842–0.904)	44,163.0
6 days	−0.068***	0.018	0.687 (0.663–0.712)	
9 days	−0.109***	0.018	0.659 (0.636–0.693)	
12 days	−0.303***	0.018	0.543 (0.524–0.563)	
**Cost (CNY)**				
20,000	0.011	0.016	Reference	
40,000	0.064***	0.016	1.055 (1.023–1.088)	
60,000	−0.016	0.015	0.974 (0.945–1.004)	
80,000	−0.059***	0.015	0.933 (0.933–0.962)	
**Medical insurance reimbursement**
0	−0.518***	0.022	Reference	
20%	−0.312***	0.021	1.229 (1.180–1.280)	−8,928.7
40%	−0.146***	0.021	1.451 (1.394–1.511)	
60%	0.138***	0.021	1.928 (1.852–2.008)	
80%	0.327***	0.020	2.329 (2.237–2.424)	
100%	0.510***	0.021	2.797 (2.685–2.914)	

#### MNL sub-group analysis

**Figure 3:**
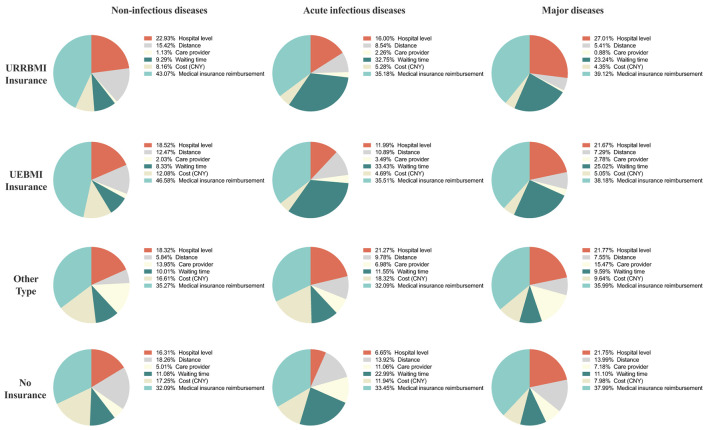
MNL results of sub-group analysis.

The result of the sub-group analysis of heterogeneity among respondents' that with different types of medical insurance has been shown in Figure 3. In the scenario of non-infectious diseases, respondents with UEBMI ranked the “reimbursement ratio” as the most important attribute, while respondents with “No insurance” considered the “reimbursement ratio” as the least important (46.58% in UEBMI vs. 43.07% in URRBMI vs. 35.27% in “other insurance types” vs. 32.09% in “no insurance”). Hospital level was considered the second most important factor except for respondents without insurance (22.93% in URRBMI vs. 18.52% in UEBMI vs. 18.32% in “Other types”).

In the scenario of acute infectious diseases, the “reimbursement ratio” remained the most important attribute among all the four insurance types. While respondents with URRBMI and UEBMI ranked the “waiting time” as the second most important attribute in their trade-off and “waiting time” weighted more in respondents with UEBMI than those with URRBMI (33.43% in UEBMI vs. 32.75% in URRBMI). “Care provider types” were treated as the least important attribute among respondents with URRBMI, UEBMI and “Other insurance types” (2.26% in URRBMI vs. 3.49% in UEBMI vs. 6.98% in “Other insurance types”).

In the scenario of major diseases, respondents still prefer the “medical insurance reimbursement ratio” the most. Respondents with URRBMI, “Other insurance types” and “No insurance” prefer “hospital level” as the second most important, while respondents with UEBMI consider “waiting time” as the second most important factor.

#### Willingness to pay

The respondents had a partiality toward tertiary hospitals (Tables 4–6). They were willing to pay CNY 2,045.0, CNY 49,040.5, and CNY 499,522.0 to transfer from private hospitals to tertiary hospitals in chronic non-communicable diseases, acute infectious diseases and major diseases, respectively. In addition, participants are willing to pay CNY 31,744.9 to shorten the distance from “across the province” to “in the county/district” when suffering from acute infectious diseases. They are also willing to pay CNY 9,149.3 to reduce waiting by 1-day. In the scenario of major diseases, participants were most willing to pay CNY 140,446.1 and CNY 44,163.0/day to obtain the shortest medical distance and 1 less day of waiting.

#### Scenario analysis

For better illustration, in Table 7, we simulated the respondents' choices and predicted their probability of choosing different levels of hospitals in different scenarios. All scenarios were analyzed under base scenarios (scenario 1 and scenario 4), which reflect the fundamental status of tertiary hospitals and community hospitals, with the probability of respondents choosing tertiary hospitals and community hospitals of 84.4 and 67.8%, respectively. Since respondents show strong partiality for tertiary hospitals, we tried to reduce the insurance reimbursement rates in tertiary hospitals, and the probability reduced to 71.2% (scenario 3 vs. scenario 1). At the same time, an 80% increase in reimbursement rates in community hospitals will bring significant improvement, with the probability of being selected increased to 76.7% (scenario 5 vs. scenario 4). This probability could reach 78.8% when the rates increase to 100%, well beyond the probability of choosing a tertiary hospital with fewer reimbursement rates (scenario 6 vs. scenario 3). Besides scenario 2, 7–12 illustrated that change of care providers and cost reduction does not affect respondents' choices.

**Table 7:** Scenario analysis^*^.

	**Base scenario**			**Base scenario**								
	**Scenario 1**	**Scenario 2**	**Scenario 3**	**Scenario 4**	**Scenario 5**	**Scenario 6**	**Scenario 7**	**Scenario 8**	**Scenario 9**	**Scenario 10**	**Scenario 11**	**Scenario 12**
Hospital level	Tertiary hospital	Tertiary hospital	Tertiary hospital	Community hospital	Community hospital	Community hospital	Community hospital	Community hospital	Community hospital	Community hospital	Community hospital	Community hospital
Distance	Within the city	Within the city	Within the city	Within the county/district	Within the county/district	Within the county/district	Within the county/district	Within the county/district	Within the county/district	Within the county/district	Within the county/district	Within the county/district
Care provider	Attending physician	Attending physician	Attending physician	Resident	Resident	Resident	Attending physician	Attending physician	Attending physician	Attending physician	Attending physician	Attending physician
Waiting time	3 days	3 days	3 days	No need to wait	No need to wait	No need to wait	No need to wait	No need to wait	No need to wait	No need to wait	No need to wait	No need to wait
Cost (CNY)	600	900	600	600	600	600	600	600	600	300	300	300
Medical insurance reimbursement	60%	60%	40%	60%	80%	100%	60%	80%	100%	60%	80%	100%
Preference	84.4%	80.5%	71.20%	67.8%	76.7%	78.8%	64.9%	75.2%	77.5%	66.1%	75.5%	77.5%

* Since our fundamental purpose is to provide policy makers with advice on how to allocate medical resources reasonably, it is necessary to consider the actual situation of China 's current medical policy. Considering the adjustability of medical policy and the impact of each attribute described above on the choice of respondents, this study mainly simulates the changes in the proportion of medical insurance reimbursement in the scenario of chronic non-infectious diseases. The reason why chronic non-infectious diseases scenario was chosen is that the biggest challenge facing the full implementation of Hierarchical Medical Treatment System in China is how to drain patients with chronic non-infectious diseases from tertiary hospitals to community hospitals. The ultimate goal is to make patients who really need high-level medical services get timely treatment. The reason for choosing the medical insurance reimbursement as the main research object is on the one hand because of its dominant position in preference analysis, and on the other hand because of its adjustability. By contrast, the inflexibility of the attributes of “distance” and “waiting time” makes them insufficient to provide a theoretical basis for policy adjustments. Moreover, “Care provider” and “Cost” have little impact on respondents' choices.

### Latent class model (LC)

**Figure 4:**
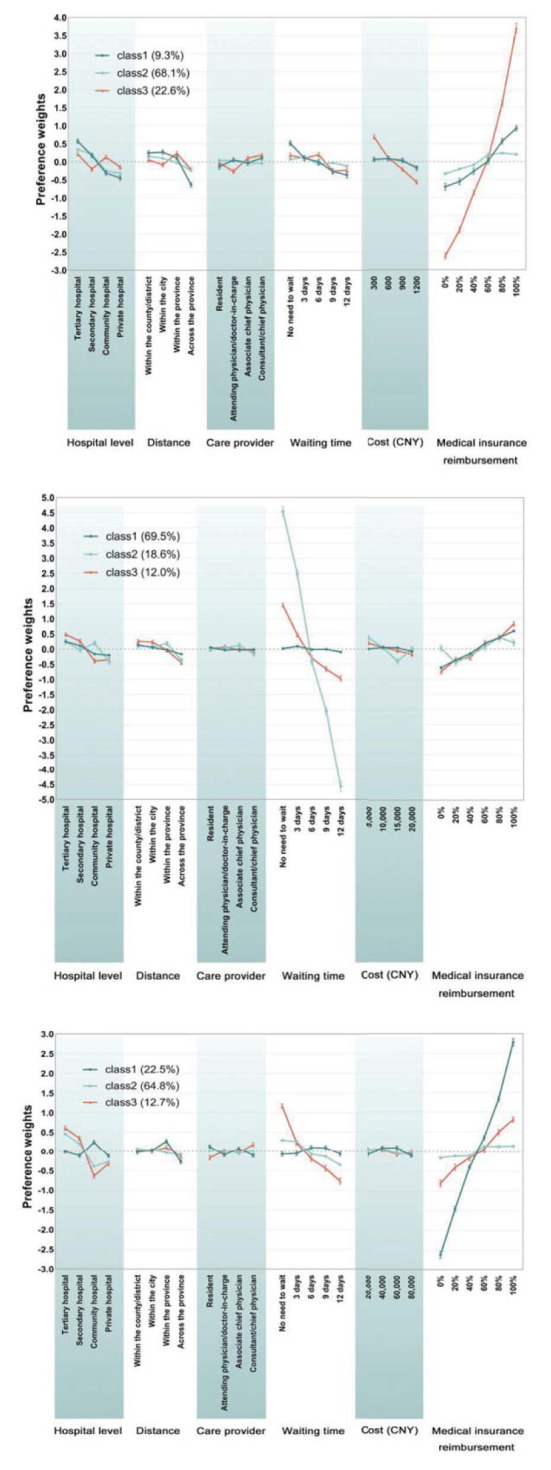
Preference weights (LC). *Images from top to bottom are non-infectious diseases scenario, acute infectious diseases scenario and major diseases scenario.

**Figure 5:**
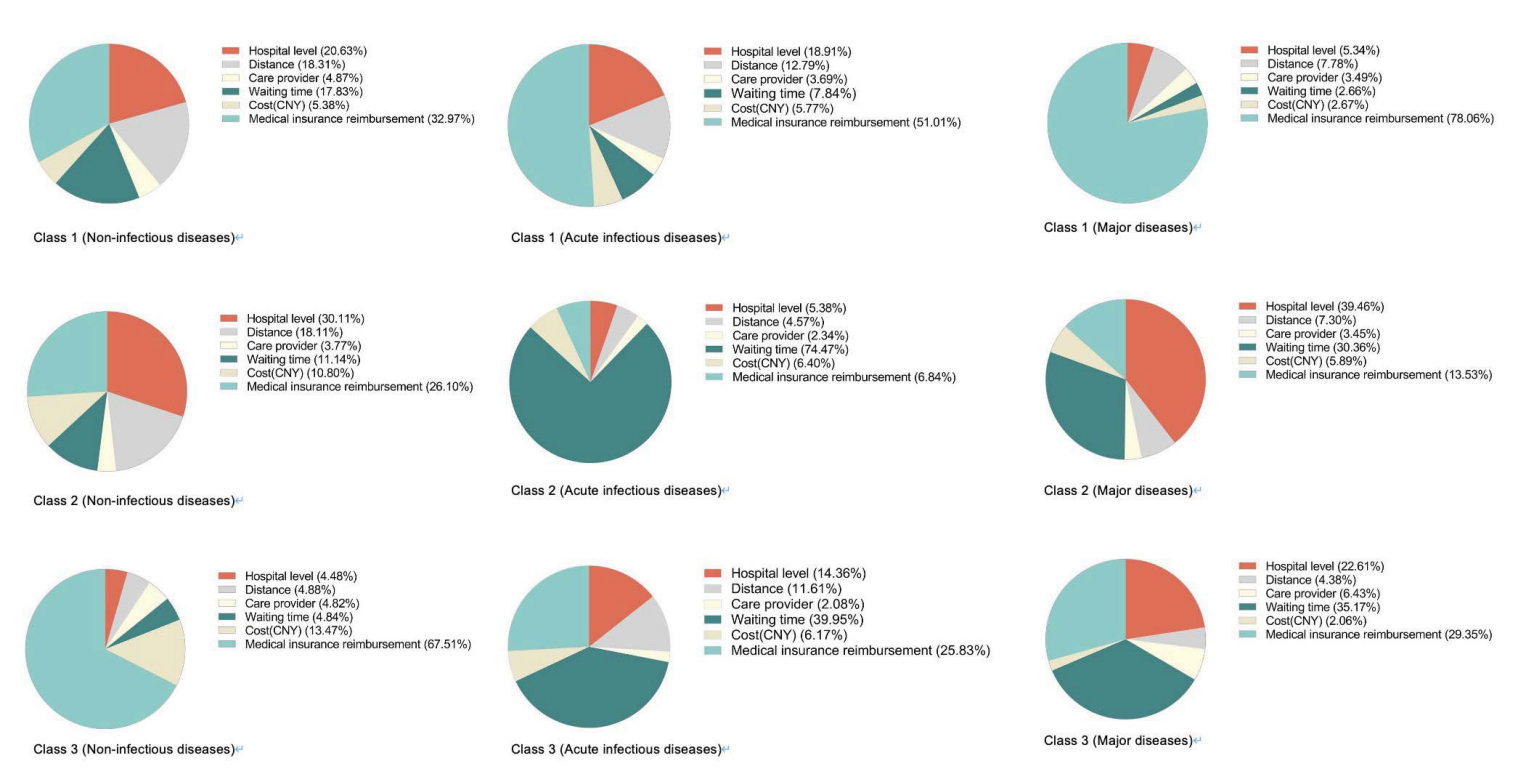
Attribute importance of LC model.

According to the previous description, the LC model with three classes is considered the most matching model. Compared with the logit model, the LC model outputs data grouped according to preference heterogeneity. The results of the LC model are shown in Tables 8–10, and people's preference weights toward different levels in the LC model have been shown in Figure 4. Figure 5 shows the preference weight of each level from the LC model. Similar to the logit model, differences of in-between utility levels are represented by vertical distance. Below is a detailed explanation of the output of the LC model for chronic non-communicable diseases scenario, and the other two scenarios are described in Appendix 2.

**Table 8:** Attributes and levels of chronic non-infectious diseases (LC).

	**Class 1 (*****n*** = **316%)**				**Class 2 (*****n*** = **2,631%)**				**Class 3 (*****n*** = **894%)**			
**Attributes levels**	**Coefficient**	**Standard error**	**OR (95%CI)**	**WTP (CNY)**	**Coefficient**	**Standard error**	**OR (95%CI)**	**WTP (CNY)**	**Coefficient**	**Standard error**	**OR (95%CI)**	**WTP (CNY)**
**Hospital level**	
Tertiary hospital	0.575^***^	0.058	Reference	Reference	0.344^***^	0.019	Reference	Reference	0.217^***^	0.050	Reference	Reference
Secondary hospital	0.176^**^	0.061	0.671 (0.596–0.756)	1,470.8	0.218^***^	0.018	0.882 (0.852–0.914)	609.4	-0.203^***^	0.051	0.657 (0.594–0.727)	299.2
Community hospital	-0.308^***^	0.066	0.414 (0.364–0.471)	3,257.1	-0.247^***^	0.019	0.554 (0.534–0.574)	2,872.6	0.134^*^	0.052	0.920 (0.831–1.018)	59.3
Private hospital	-0.443^***^	0.067	0.362 (0.317–0.412)	3,755.1	-0.315^***^	0.018	0.517 (0.499–0.536)	3,201.6	-0.148^**^	0.051	0.694 (0.627–0.767)	260.5
**Distance**	
Within the county/district	0.250^***^	0.061	Reference	Reference	0.158^***^	0.019	Reference	Reference	0.055	0.052	Reference	Reference
Within the city	0.270^***^	0.060	1.020 (0.906–1.148)	-995.9	0.100^***^	0.019	0.943 (0.909–0.979)	-485.3	-0.078	0.052	0.875 (0.790–0.970)	55.8
Within the province	0.114	0.061	0.873 (0.774–0.984)	503.1	-0.020	0.018	0.837 (0.808–0.867)	866.3	0.241^***^	0.048	1.205 (1.096–1.325)	-132.7
Across the province	-0.633^***^	0.069	0.414 (0.361–0.474)	3,259.1	-0.238^***^	0.018	0.673 (0.649–0.698)	1,925.3	-0.217^***^	0.051	0.762 (0.689–0.843)	193.6
**Care provider**	
Resident	-0.133^*^	0.062	Reference	Reference	0.043^*^	0.018	Reference	Reference	-0.030	0.049	Reference	Reference
Attending physician	0.057	0.061	1.209 (1.072–1.364)	-700.5	0.035	0.019	0.993 (0.957–1.029)	35.3	-0.263^***^	0.051	0.793 (0.717–0.875)	165.6
Associate chief physician	-0.030	0.063	1.109 (0.981–1.253)	-380.6	-0.040^*^	0.019	0.921 (0.888–0.955)	401.2	0.103	0.053	1.143 (1.030–1.268)	−95.1
Consultant/chief physician	0.107	0.062	1.271 (1.127–1.435)	-886.4	-0.038^*^	0.018	0.922 (0.890–0.956)	393.4	0.189^***^	0.051	1.245 (1.127–1.375)	−156.1
**Waiting time**												
No need to wait	0.517^***^	0.067 0.071	Reference	270.4	0.071^***^	0.021	Reference	75.0	0.185^**^	0.060	Reference	24.8
3 days	0.104		0.662 (0.575–0.761)		0.130^***^	0.022	1.060 (1.060 -1.107)		0.098	0.059	0.916 (0.816–1.028)	
6 days	0.003	0.073	0.599 (0.519–0.691)		-0.065^**^	0.022	0.873 (0.837–0.911)		0.202^***^	0.060	1.017 (0.904–1.143)	
9 days	-0.261^***^		0.460 (0.397–0.532)		-0.022	0.021	0.911 (0.873–0.950)		-0.252^***^	0.062	0.646 (0.572–0.729)	
12 days	-0.363^***^	0.075 0.077	0.415 (0.357–0.482)		-0.114^***^	0.022	0.831 (0.796–0.867)		-0.233^***^	0.059	0.658 (0.586–0.739)	
Cost (CNY)
300	0.062	0.061	Reference		0.044^*^	0.018	Reference		0.688^***^	0.056	Reference	
600	0.084	0.062	1.022 (0.906–1.153)		0.095^***^	0.019	1.052 (1.015–1.091)		0.105^*^	0.051	0.558 (0.505–0.617)	
900	0.036	0.062	0.974 (0.863–1.100)		0.003	0.018	0.960 (0.926–0.995)		-0.218^***^	0.051	0.404 (0.366–0.447)	
1,200	-0.182^**^	0.064	0.784 (0.784–0.888)		-0.141^***^	0.018	0.831 (0.831–0.861)		-0.575^***^	0.052	0.283 (0.283–0.313)	
**Medical insurance reimbursement**	
0	−0.696^***^	0.094	Reference		−0.333^***^	0.025	Reference	−26.0	−2.620^***^	0.085	Reference	
20%	−0.549^***^	0.090	1.158 (0.970–1.383)	−60.0	−0.205^***^	0.024	1.137 (1.083–1.193)		−1.902^***^	0.074	2.052 (1.774–2.372)	−45.1
40%	−0.267^**^	0.085	1.536 (1.301–1.813)		−0.089^**^	0.025	1.277 (1.217–1.340)		−0.871^***^	0.063	5.751 (5.078–6.513)	
60%	0.014	0.080	2.035 (1.740–2.381)		0.187^***^	0.025	1.683 (1.603–1.768)		0.060	0.064	14.594 (12.866–16.554)	
80%	0.569^***^	0.075	3.542 (3.057–4.104)		0.237^***^	0.025	1.770 (1.686–1.857)		1.622^***^	0.074	69.535 (60.114–80.433)	
100%	0.929^***^	0.074	5.082 (4.395–5.876)		* 0.203^***^	0.025	1.710 (1.629–1.794)		3.710^***^	0.127	561.425 (437.625–720.247)	
**Influence Factors**	***N***	**%**	***P*** **valve**		***N***	**%**	***P*** **valve**		***N***	***%***	***P*** **valve**	
**Sex**						0.000				0.000	
Male	95	4.966	Reference		1,303	68.113			515	26.921	
Female	218	11.378			1,320	68.894			378	19.727	
Other	3	25.000			8	66.667			1	8.333	
**Age**						0.140			0.000	
Under 18	19	13.103			108	74.483			18	12.414	
18–25	143	9.610			1,095	73.589			250	16.801	
26–35	80	7.181			784	70.377			250	22.442	
36–45	40	6.462			379	61.228			200	32.310	
46–55	23	8.303			143	51.625			111	40.072	
56–60	7	6.604			53	50.000			46	43.396	
Above 60	4	4.348			69	75.000			19	20.652	
**Educational Background**						0.107				0.069	
Middle School education or below	21	6.052			239	68.876			87	25.072	
High School education	51	9.358			364	66.789			130	23.853	
Vocational school education	54	6.725		541	67.372		208	25.903	
Bachelor's degree	173	9.222			1,281	68.284			422	22.495	
Master's degree	15	6.122			186	75.918			44	17.959	
PhD degree	2	8.000			20	80.000			3	12.000	
**Registered residence**						0.054				0.012	
Rural	174	7.433			1,601	68.390			566	24.178	
Urban	142	9.467			1,030	68.667			328	21.867	
**Monthly Income (CNY)**						0.000				0.000	
Under 5,000	185	10.638			1,221	70.213			333	19.149	
5,000–10,000	101	6.336			1,059	66.437			434	27.227	
10,001–20,000	25	5.841			297	69.393			106	24.766	
More than 20,000	3	4.225			50	70.423			18	25.352	
**Insurance type**						0.780				0.001	
URRBMI	203	8.807			1,617	70.152			485	21.041	
UEBMI	99	7.164			894	64.689			389	28.148	
Other Commercial Insurance	10	9.615			82	78.846			12	11.538	
No Insurance	4	8.000			38	76.000			8	16.000	

*** *p* < 0.001

** *p* < 0.01, and

* *p* < 0.05.

**Table 9:** Attributes and levels of acute infectious diseases (LC).

	**Class 1 (*****n*** = **2,668%)**				**Class 2 (*****n*** = **731%)**				**Class 3 (*****n*** = **442%)**			
**Attributes levels**	**Coefficient**	**Standard error**	**OR (95%CI)**	**WTP (CNY)**	**Coefficient**	**Standard error**	**OR (95%CI)**	**WTP (CNY)**	**Coefficient**	**Standard error**	**OR (95%CI)**	**WTP (CNY)**
**Hospital level**												
Tertiary hospital	0.249^***^	0.019	Reference	Refere nce	0.243^**^	0.080	Reference	Reference	0.480^***^	0.055	Reference	Reference
Secondary hospital	0.114^***^	0.018	0.874 (0.843–0.905)	23,798.7	-0.024	0.073	0.766 (0.664–0.883)	10,638.7	0.260^***^	0.057	0.802 (0.717–0.898)	8,820.7
Community hospital	-0.162^***^	0.019	0.663 (0.639–0.688)	72,332.5	0.197^**^	0.073	0.955 (0.827–1.102)	1,845.5	-0.391^***^	0.063	0.419 (0.370–0.474)	34,9078
Private hospital	-0.201^***^	0.018	0.638 (0.616–0.662)	79,1560	-0.416^***^	0.075	0.518 (0.446–0.600)	26,2761	-0.349^***^	0.062	0.437 (0.387–0.493)	33,2235
**Distance**	
Within the county/district	0.138^***^	0.019	Reference	Reference	0.105	0.073	Reference	Reference	0.256^***^	0.058	Reference	Reference
Within the city	0.055^**^	0.019	0.920 (0.887–0.955)	−9,677.1	0.083	0.079	0.978 (0.838–1.142)	−3,314.7	0.232^***^	0.057	0.976 (0.872–1.093)	-9,297.0
Within the province	-0.027	0.018	0.848 (0.818–0.879)	29,064.4	0.185^*^	0.074	1.084 (0.938–1.252)	−3,201.4	-0.040	0.059	0.744 (0.663–0.835)	11,842.4
Across the province	-0.166^***^	0.018	0.738 (0.712–0.765)	53,539.4	-0.374^***^	0.075	0.620 (0.534–0.718)	19,101.9	-0.448^***^	0.063	0.494 (0.437–0.559)	28,230.7
	**Class 1 (*****n*** = **316%)**				**Class 2 (*****n*** = **2,631%)**				**Class 3 (*****n*** = **894%)**			
**Attributes levels**	**Coefficient**	**Standard error**	**OR (95%CI)**	**WTP (CNY)**	**Coefficient**	**Standard error**	**OR (95%CI)**	**WTP (CNY)**	**Coefficient**	**Standard error**	**OR (95%CI)**	**WTP (CNY)**
**Care provider**	
Resident	-0.055^**^	0.018	Reference	Reference	0.001	0.076	Reference	Reference	0.009	0.058	Reference	Reference
Attending physician	-0.032	0.019	0.916 (0.883–0.950)	15,442.	0.033 0.126	0.076	1.032 (0.890–1.197)	−1,266. 2	0.071	0.058	1.064 (0.949–1.193)	−2,496. 5
Associate chief physician	-0.014	0.019	0.933 (0.899–0.968)	7 12,248.	-0.160^*^	0.074	1.132 (0.979–1.310)	-4,957. 8	-0.026	0.059	0.966 (0.861–1.084)	1,384.0
Consultant/chief physician	-0.009	0.018	0.938 (0.905–0.973)	4 11,251.4		0.071	0.851 (0.740–0.978)	6,448.0	-0.055	0.059	0.938 (0.835–1.054)	2,556.5
**Waiting time**	
No need to wait	0.017	0.022	Reference	1,601.4	4.541^***^	0.159	Reference	30,289.4	1.450^***^	0.061	Reference	8,092.8
3 days	0.094^***^	0.022	1.080 (1.035–1.128)		2.481^***^	0.120	0.127 (0.101–0.161)		0.473^***^	0.064	0.377 (0.332–0.427)	
6 days	-0.012	0.022	0.972 (0.931–1.014)		-0.411^***^	0.092	0.007 (0.006–0.008)		-0.293^***^	0.072	0.175 (0.152–0.201)	
9 days	-0.006	0.022	0.978 (0.937–1.020)		-2.042^***^	0.106	0.001 (0.001–0.002)		-0.658^***^	0.078	0.121 (0.104–0.141)	
12 days	-0.092^***^	0.022	0.897 (0.860–0.935)		-4.569^***^	0.154	0.000 (0.000–0.000)		-0.973^***^	0.085	0.089 (0.075–0.105)	
**Cost (CNY)**												
300	-0.001	0.019	Reference		0.374^***^	0.074	Reference		0.192^***^	0.057	Reference	
600	0.051^**^	0.019	1.053 (1.015–1.092)		0.039	0.079	0.715 (0.613–0.835)		0.049	0.059	0.867 (0.772–0.973)	
900	0.037^*^	0.018	1.039 (1.002–1.077)		-0.410^***^	0.078	0.457 (0.392–0.532)		-0.060	0.059	0.777 (0.692–0.873)	
1,200	-0.086^***^	0.018	0.918 (0.918–0.952)		-0.002	0.072	0.687 (0.687–0.791)		-0.182^**^	0.060	0.688 (0.688–0.774)	
**Medical insurance reimbursement**	
0	−0.618^***^	0.026	Reference		0.022		Reference		−0.742^***^	0.087	Reference	−628.1
20%	−0.366^***^	0.025	1.287 (1.227–1.351)	−2,134.9	−0.457^***^	0.099	0.620 (0.503–0.763)	−74.1	−0.360^***^	0.082	1.465 (1.247–1.72) 0)	
40%	−0.158^***^	0.024	1.585 (1.511–1.662)		−0.213^*^	0.106	0.791 (0.659–0.949)		−0.286^***^	0.079	1.578 (1.351–1.84)3)	
60%	0.173^***^	0.025	2.206 (2.102–2.316)		0.060	0.093	1.038 (0.842–1.281)		0.199^**^	0.075	2.561 (2.211–2.96)6)	
80%	0.374^***^	0.024	2.698 (2.572–2.830)		0.380^***^	0.107	1.431 (1.180–1.737)		0.364^***^	0.074	3.021 (2.613–3.49)1)	
100%	0.594^***^	0.025	3.359 (3.196–3.530)		0.208^*^	0.099 0.098	1.204 (0.994–1.460)		0.825^***^	0.071	4.791 (4.171–5.50)2)	
**Influence factors**	***N***	**%**	***P*** **valve**	***N***	**%**	***P*** **valve**		***N***	**%**	***P*** **valve**	
**Sex**						0.345				0.000	
Male	1,396	72.974	Reference		386	20.178		131	6.848	
Female	1,266	66.075			341	17.797		309	16.127	
Other	6	50.000			4	33.333			2	16.667	
**Age**						0.000				0.007	
Under 18	113	77.931			23	15.862		9	6.207	
18–25	1,056	70.968			223	14.987		209	14.046	
26–35	792	71.095			186	16.697		136	12.208	
36–45	446	72.052			120	19.386		53	8.562	
46–55	177	63.899			74	26.715		26	9.386	
56–60	58	54.717			41	38.679		7	6.604	
Above 60	26	28.261		64	69.565		2	2.174	
**Educational background**						0.000				0.000	
Middle School education or below	217	62.536		109	31.412		21	6.052	
High School education	388	71.193		95	17.431		62	11.376	
Vocational school education	603	75.093		132	16.438		68	8.468	
Bachelor's degree	1,208	66.962		344	19.069		252	13.969	
Master's degree	162	66.122		47	19.184		36	14.694	
PhD degree	18	72.000		4	16.000		3	12.000	
**Registered Residence**						0.007				0.1	
Rural	1,611	68.817		482	20.589		248	10.594	
Urban	1,057	70.467		249	16.600		194	12.933	
**Monthly income (CNY)**						0.002				0.000	
Under 5,000	1,127	64.807		366	21.047		246	14.146	
5,000–10,000	1,158	72.647		273	17.127		163	10.226	
10,001–20,000	323	75.467		75	17.523		30	7.009	
More than 20,000	54	76.056		15	21.127		2	2.817	
**Insurance type**						0.142				0.367	
URRBMI	1,595	69.197		432	18.742		278	12.061	
UEBMI	958	69.320		276	19.971		148	10.709	
Other Commercial Insurance	83	79.808		12	11.538		9	8.654	
No Insurance	32	64.000		11	22.000		7	14.000	

*** *p* < 0.001

** *p* < 0.01, and

* *p* < 0.05.

**Table 10:** Attributes and levels of major diseases (LC).

	**Class 1 (*****n*** = **881%)**				**Class 2 (*****n*** = **2,499%)**				**Class 3 (*****n*** = **461%)**			
**Attributes levels**	**Coefficient**	**Standard error**	**OR (95%CI)**	**WTP (CNY)**	**Coefficient**	**Standard error**	**OR (95%CI)**	**WTP (CNY)**	**Coefficient**	**Standard error**	**OR (95%CI)**	**WTP (CNY)**
**Hospital level**												
Tertiary hospital	−0.007	0.047	Reference	Reference	0.455^***^	0.019	Reference	Reference	0.607^***^	0.052	Reference	Reference
Secondary hospital	−0.116^*^	0.046	0.897 (0.819–0.982)	194,066.	0.206^***^	0.018	0.780	164,839.2	0.347^***^	0.053	0.771 (0.694–0.856)	264,576.3
Community hospital	0.247^***^	0.048	1.289 (1.173–1.417)	−453,60	−0.388^***^	0.019	0.431 (0.414–0.447)	57,617.8	−0.636^*^	0.064	0.288 (0.255–0.327)	1,262,09
Private hospital	−0.123^*^	0.047	0.891 (0.812–0.977)	206,599.1	−0.272^***^	0.019	0.483 (0.466–0.502)	480,957.6	0.059	0.396 (0.353–0.445)	939,470.5	
**Distance**	
Within the county/district	−0.026	0.048	Reference	Reference	0.075^***^	0.019	Reference	Reference	0.019	0.056	Reference	Reference
Within the city	0.027	0.049	1.054 (0.958–1.161)	−48,469.3	0.046^*^	0.019	0.972 (0.935–1.009)	−30,635.6	0.001	0.056	0.982 (0.881–1.096)	−1,232.6
Within the province	0.269^***^	0.045	1.342 (1.228–1.467)	−525,489.3	−0.041^*^	0.019	0.891 (0.859–0.924)	76,587.0	0.110^*^	0.055	1.095 (0.984–1.220)	−92,494.2
Across the province	−0.270^***^	0.049	0.784 (0.712–0.862)	435,554.2	−0.081^***^	0.019	0.856 (0.825–0.888)	103,219.1	−0.130^*^	0.056	0.861 (0.771–0.961)	151,772.9
	**Class 1 (*****n*** = **316%)**				**Class 2 (*****n*** = **2,631%)**				**Class 3 (*****n*** = **894%)**			
**Attributes levels**	**Coefficient**	**Standard error**	**OR (95%CI)**	**WTP (CNY)**	**Coefficient**	**Standard error**	**OR (95%CI)**	**WTP (CNY)**	**Coefficient**	**Standard error**	**OR (95%CI)**	**WTP (CNY)**
**Care provider**	
Resident	0.130^**^	0.046	Reference	Reference	0.016	0.019	Reference	Reference	−0.169^**^	0.057	Reference	Reference
Attending physician	−0.099^*^	0.046	0.795 (0.726–0.871)	409,158.5	0.024	0.019	1.009 (0.972–1.047)	−5,799.6	0.013	0.055	1.199 (1.076–1.337)	−184,229.4
Associate chief physician	0.080	0.048	0.951 (0.866–1.044)	90,355.7	−0.049^*^	0.019	0.937 (0.903–0.973)	43,009.0	−0.029	0.056	1.150 (1.031–1.283)	−142,104.4
Consultant/chief physician	−0.111^*^	0.048	0.786 (0.715–0.863)	430,817.1	0.009	0.019	0.994 (0.958–1.031)	4,030.4	0.185^***^	0.055	1.425 (1.280–1.585)	−359,198.0
**Waiting time**	
No need to wait	−0.078	0.056	Reference	−1,052.2	0.297^***^	0.022	Reference	35,748.3	1.168^***^	0.058	Reference	163,650.4
3 days	−0.057	0.056	1.020 (0.915–1.138)		0.264^***^	0.023	0.968 (0.926–1.011)		0.233^***^	0.062	0.393 (0.348–0.444)	
6 days	0.106	0.056	1.202 (1.078–1.341)		−0.075^***^	0.022	0.689 (0.660–0.720)		−0.195^**^	0.067	0.256 (0.225–0.292)	
9 days	0.100	0.056	1.194 (1.070–1.332)		−0.135^***^	0.022	0.649 (0.621–0.678)		−0.439^***^	0.070	0.201 (0.175–0.230)	
12 days	−0.071	0.055	1.007 (0.905–1.121)		−0.351^***^	0.022	0.523 (0.501–0.547)		−0.767^***^	0.076	0.144 (0.125–0.168)	
**Cost (CNY)**	
300	−0.066	0.048	Reference		0.032	0.019	Reference		0.036	0.054	Reference	
600	0.081	0.047	1.158 (1.055–1.271)		0.067^***^	0.019	1.036 (0.998–1.076)		0.051	0.056	1.015 (0.910–1.132)	
900	0.085	0.047	1.164 (1.061–1.276)		−0.040^*^	0.019	0.931 (0.897–0.966)		−0.063	0.056	0.906 (0.812–1.012)	
1,200	−0.100^*^	0.047	0.967 (0.967–1.060)		−0.059^**^	0.019	0.913 (0.913–0.948)		−0.024	0.056	0.943 (0.943–1.052)	
**Medical insurance reimbursement**	
0	−2.623^***^	0.087	Reference	−96,484.0	−0.157^***^	0.026	Reference	−1,912.4	−0.802^***^	0.086	Reference	−16,386.8
20%	−1.455^***^	0.065	3.219 (2.836–3.653)		−0.111^***^	0.025	1.047 (0.996–1.100)		−0.393^***^	0.079	1.506 (1.289–1.759)	
40%	−0.383^***^	0.057	9.394 (8.395–10.512)		−0.108^***^	0.025	1.050 (0.999–1.104)		−0.155^*^	0.074	1.910 (1.653–2.207)	
60%	0.345^***^	0.059	19.461 (17.328–21.856)		0.124^***^	0.026	1.324 (1.259–1.392)		0.051	0.072	2.347 (2.037–2.703)	
80%	1.336^***^	0.063	52.417 (46.365–59.259)		0.121^***^	0.025	1.320 (1.256–1.387)		0.489^***^	0.069	3.637 (3.178–4.162)	
100%	2.781^***^	0.092	222.371 (185.601–266.425)		0.132^***^	0.025	1.335 (1.270–1.403)		0.812^***^	0.067	5.025 (4.410–5.725)	
**Influence Factors**	***N***	**%**	***P*** **valve**		**N**	**%**	***P*** **valve**	***N***	***%***	***P valve***	
**Sex**						0.716				0.000	
Male	469	24.516	Reference		1,300	67.956		144	7.527	
Female	409	21.347			1,193	62.265		314	16.388	
Other	3	25.000			6	50.000		3	25.000	
**Age**						0.000				0.001	
Under 18	25	17.241			108	74.483		12	8.276	
18–25	321	21.573			958	64.382		209	14.046	
26–35	264	23.699			710	63.734		140	12.567	
36–45	184	29.725			382	61.712		53	8.562	
46–55	65	23.466			176	63.538		36	12.996	
56–60	16	15.094			81	76.415		9	8.491	
Above 60	6	6.522			84	91.304		2	2.174	
**Educational background**						0.000				0.000	
Middle School education or below	62	17.867		260	74.928		25	7.205	
High School education	118	21.651		359	65.872		68	12.477	
Vocational school education	230	28.643		507	63.138		66	8.219	
Bachelor's degree	411	21.908		1,195	63.699		270	14.392	
Master's degree	57	23.265		158	64.490		30	12.245	
PhD degree	3	12.000		20	80.000		2	8.000	
**Registered residence**						1.000				0.474	
Rural	539	23.024		1,530	65.357		272	11.619	
Urban	342	22.800		969	64.600		189	12.600	
**Monthly income (CNY)**						0.000				0.000	
Under 5,000	316	18.171		1,165	66.993		258	14.836	
5,000−10,000	429	26.913		1,001	62.798		164	10.289	
10,001–20,000	118	27.570		275	64.252 71.831		35	8.178	
More than 20,000	17	23.944		51			3	4.225	
**Insurance type**						0.119				0.400	
URRBMI	509	22.082		1,512	65.597		284	12.321	
UEBMI	344	24.891		879	63.603		159	11.505	
Other commercial insurance	18	17.308		75	72.115		11	10.577	
No Insurance	10	20.000		33	66.000		7	14.000	

*** *p* < 0.001

** *p* < 0.01, and

* *p* < 0.05.

#### Chronic non-infectious diseases

For the respondents of class 1, “medical insurance reimbursement” is the foremost consideration, accounting for 33.0%, followed by “hospital level” (20.6%), “waiting time” (17.8%), and “distance” (18.3%). The utility of “medical insurance reimbursement” has changed significantly, from “0%” to “100%” (increased by 1.63). The utility spans of attributes “hospital level,” “waiting time,” and “distance” are 1.01, 0.88, and 0.87, respectively. Overall, “100%,” “80%,” “tertiary hospital,” and “no need to wait” are preferable. When “tertiary hospital” is used as a reference level, the odds ratio of other levels is < 1, which indicates that “tertiary hospital” is the preferred choice for such respondents. In the “distance” attribute with “within the country/district” as the reference level, the odds ratio of “within the city” is 1.020 (95% CI 0.906–1.148), indicating that respondents are slightly more inclined to choose hospitals in the city, which may be related to the allocation of medical facilities in the areas where they live. In contrast to medical reimbursement, the odds ratio decreased as the waiting time increased, and “CNY 1,200” is the most undesirable choice. People in this class have more comprehensive considerations.

The attribute of “hospital level” is most valued by respondents of class 2, with its importance accounting for 30.1%, higher than “medical insurance reimbursement,” which accounts for 26.1%. Differences of preference weights in between levels also indicate the importance of attributes. Positive/negative and the coefficient magnitude show the respondents' preference for each level. In this case, the desired levels are “tertiary hospital,” “80,” and “100%.” Participants tend to pursue tertiary hospitals and shorter travel distances.

Interestingly, in the “distance” attribute, respondents seemed to prefer the “3 days” level compared to “no waiting time.” As for medical cost and reimbursement rates, respondents preferred “CNY 600” and “80%” rather than the reference level in which they pay less. This may be related to conventional thought, as some people think price and service quality are closely linked. Compared with class 1, people in this class are inclined to seek higher-quality medical services.

Respondents of class 3 mainly focused on higher “medical insurance reimbursement,” with the importance accounting for 67.5%, followed by “cost” (13.5%). As for “medical insurance reimbursement” attributes, utility increased significantly by 6.3% with the levels change. Not surprisingly, this type specifically focused on the highest reimbursement rates, and the odds ratio of level “100%” was 561.425 (437.625–720.247). Compared with the first two classes, respondents in this class did not seem to care about the hospital level or waiting time but about the more cost-effective medical services.

## Discussion

This study is the first quantitative analysis of Chinese residents' healthcare preferences under different types of diseases. Although some scholars have analyzed the healthcare preferences of Chinese residents with chronic diseases (37–40), no study categorized the diseases into three separate types at the same time and analyzed them in two models. Particularly, medical insurance reimbursement as an attribute is also a crucial novel addition in this study. There may be confusing about healthcare service costs and medical insurance reimbursement, believing that the actual impact is self-paid costs. Theoretically, the patients may attach more importance to the factor of medical out-of-pocket medical expenses. While in fact, for the general public, medical insurance reimbursement has an impact not only on the issue of out-of-pocket costs, but also, to a certain extent, correlated with residents' trust, respect, and their approbation degree to hospitals. Rises in the reimbursement rates reduce patients' self-paid costs and represent the recognition from the government because favorable policies are often formulated after a trade-off between advantages and disadvantages by the government. In addition, during our semi-structured discussion with experts and residents, we found that even with the same out-of-pocket expenses, residents would still choose to go to a hospital with a higher medical insurance reimbursement ratio. They might believe that the government's financial support for a hospital is, to some extent, an affirmation of its medical ability. Also, the attribute of medical reimbursement ratio provides us with insights that how will people's preference change according to the changes in reimbursement ratio. Additionally, our study results substantiates that compared with healthcare service costs, the medical insurance reimbursement has a far more enlightening impact on their preference and choice (41).

The preference heterogeneity was analyzed through two widely-used models. MNL can help investigate the preference heterogeneity of the whole population, and the LC model applies for determining which treatment or health policy implementation is most effective in a specific population. The results of the MNL model show that “medical insurance reimbursement” occupies an absolute advantage in chronic non-communicable diseases, accounting for 45.0%, followed by “hospital level” and “distance.” In acute illness, respondents value the attribute of “waiting time” as much as “medical insurance reimbursement,” which indicates that hospitals with effective healthcare services will be preferable. Since respondents hoped to get treatment as soon as possible, levels of medical facilities became relatively less important. For critical diseases, preference for tertiary hospitals with better medical resources showed again, while the most important attribute is still medical insurance reimbursement. Like the acute disease situation, attention to waiting time cannot be ignored. This suggests that people want to get rapid and high-quality medical services when suffering from major diseases. Overall, medical insurance reimbursement carries the most weight, providing enlightening ideas for related policy intervention toward the welldeveloped system.

Meanwhile, the results of the sub-group MNL analysis are highly similar to the results of the general MNL analysis. And moreover, slight differences were found in the preference of respondents with different medical insurance types. Such results may be closely related to the nature of the URRBMI and UEBMI. People with UEBMI are required to seek medical services at medical insurance-designated medical institutions before they can enjoy the benefits of reimbursement, which leaves limited options for these people to choose the hospital level (42), while for those with URRBMI can choose different hospitals without the restriction of hospital level during different age periods. Meanwhile, the payment of UEBMI is borne according to the joint contribution of the employers and the employee, while URRBMI is paid by individuals through their community committee or schools, with subsidies provided by the government. The payment standards vary a lot for different age periods. Additionally, URRBMI-insured individuals can choose the payment standard according to their financial ability (42, 43).

Scenario analysis can deeply explore how the target population makes choices in a risk-benefit balance and how the level of a particular attribute change affects these choices. Our results show that although people strongly prefer tertiary hospitals, this is not unchangeable. Changes in reimbursement rates could make a difference. Hospital levels and waiting times fail to affect utility because Chinese patients are more concerned with hospitals than with doctors, especially in public hospitals. People's distrust of community hospitals may be due to the uneven distribution of medical resources. While remarkable achievements have been made in China, especially in reducing mortality and prolonging life expectancy (44), uneven distribution of medical resources has been the main concern in recent years, especially in rural areas. Statistics have shown that as of 2019, the proportion of medical staff who has obtained a bachelor's or higher degree in the village clinic or township health centers is <35.0%.

Meanwhile, the professor or associate professor accounts for more than 10% of the medical staff in hospitals, approximately double the proportion in township CHCs and four times that in village clinics. Besides, in 2019, the number of equipment worth over 10,000 RMB in hospitals is nearly 10-times the number in CHCs, and the number of equipment worth over 1 million RMB in hospitals is more than 20-times the number in CHCs (9). While the existing policy is based on population density to allocate medical resources, studies have shown that the economically and technologically advantaged areas, mainly the eastern regions, showed the most redundancies of medical input that have not been fully utilized (45). However, improvements in facilities and services of community hospitals alone may not completely solve the problem.

Systematic healthcare reformation can achieve wider coverage in the Chinese health agenda. To accelerate the process, political support has been proven to be an essential factor (46). Apart from the increment in medical infrastructures, which requires a large amount of capital, the government should consider public preference and potential behavioral intentions for a more realistic plan to yield the greatest returns on medical budgets.

Another phenomenon is that private hospitals are less likely to be chosen when people seek medical opinions. This may be due to the lack of confidence in these medical institutions. The low social trust may come from old stereotypes because only a few medical technologies have administrations before 2009 (47). People are more willing to receive treatment in general hospitals funded by the government, where they can receive in-patient management and benefit from a high-quality referral system. Besides, most private hospitals are located in developed areas (48), so people in rural areas may not be familiar with these hospitals. However, scholars have found that although the number of employees in some private hospitals is less than in public hospitals, the level of vocational qualification certificates is higher than that of public hospital employees (49).

Moreover, according to previous studies, the quality of medical services is not directly related to the type of ownership (50), since no statistical difference was found in the mortality rates between general hospitals and private hospitals of the same scale. In this case, many researchers have advocated the health policy to enable more equitable access to private care (48, 51). In addition to community hospitals, private hospitals may also become medical facilities for sharing patients in tertiary hospitals, which is worth considering by the government.

From the results of this study, to accelerate the process of hierarchical diagnosis and treatment, the government and health departments are suggested to attach more importance on medical reimbursement rates and the disparities in reimbursement ratios among regions and among different healthcare insurance types. Although in some regions of China, the reimbursement ratio of medical insurance is already very high, inequity still exists due to the nature of individuals, regions, or even healthcare insurance types. Consideration could also be given to the appropriate reallocation of funds, originally subsidies, of the community hospital to improve the level of healthcare services and to increase reimbursement rates. Besides, reducing reimbursement rates in tertiary hospitals while increasing those of community hospitals may also help achieve this goal. This action will be fruitful, especially for attracting chronic patients to transfer to community hospitals. Meanwhile, accelerating the process of a diagnosis-related groups (DRGs) payment system is another available measure, not only to improve the current practice of hierarchical diagnosis and treatment system but also to help avoid the moral hazard issue, which could have hindered the development of the medical system in China, raised between clinicians and patients. This study divided diseases into three types, while future scholars can conduct studies on preference from the detailed diseases to formulate more rigorous policies.

### Comparison with previous studies worldwide

Current studies worldwide mainly focus on structure attributes and process attributes. For example, we found three studies (52–54) which conducted DCE and found that waiting time till the appointment as the most important attribute. Four studies (55–58) found the attribute of “Care provider” or “Professional person” as the most preferred one, i.e., doctor *vs*. practice nurse, compared with waiting time, and the likelihood of having illness cured, etc. Several studies focus on the attribute of “attention paid by professionals” (14, 59). Moreover, there are two studies (60, 61) that found the outcome attribute to be the most important attribute, i.e., the probability of receiving high-quality treatment. Compared with these previous studies, our study has both consistent and contradictory results, e.g., waiting time accounts large proportion of importance weights in both acute infectious diseases and major diseases. While the “care provider” attribute was found to be relatively less important in our study. At the same time, our study found the medical insurance reimbursement ratio to be the absolute most important attribute, which is a new contribution to the existing literature.

### Implications for policymaking

Rational allocation of medical resources in China cannot be achieved without equity of allocation and efficiency of allocation (62). Equity of the medical and health allocation, according to our result, still exists among various medical insurance types. The different nature of medical insurance, especially the reimbursement ratio of URRBMI and UEBMI, results in heterogeneous preferences of different insured, e.g., people with URRBMI weighted the hospital level more, compared with UEBMI insured for the potential reason of limited choice of hospital level. And this may provide insights for policymakers, especially the medical insurance system policymakers, to adjust the reimbursement ratio and restrictions of choice of hospital levels among different insurance types. This may help policymakers to better improve the current practice of the hierarchical medical system, mainly by restricting insureds' choices and preferences.

Similarly, rising medical insurance reimbursement ratios in community hospitals and reducing in tertiary hospitals both help improve the practice of the diversion of patients with mild and chronic diseases from tertiary hospitals to community hospitals. Although China has enacted relevant adjustment regulations, they are still insufficient to effectively actualize patients' diversion, which leads that patients are continuing to seek treatment at tertiary institutions subconsciously. And this may be caused by a combination of public misconceptions about their own illnesses and the structure of community hospitals, as well as a modest variation in the percentage of medical insurance coverage between hospitals of various levels, e.g., implementing the policies that allow primary hospitals to offer the elderly door-to-door or skip-the-line service, which would effectively lessen the burden of elderly outpatients on tertiary hospitals. Policymakers may start from the “waiting time” of the healthcare services and simultaneously attempt different combinations of policies to increase the convenience of health services, especially for the elderly.

### Significance and promotion of results

This study explores the healthcare preference of Chinese residents from a new perspective, medical insurance reimbursement, which has never been discussed in detail in previous studies. Besides, results showed that reimbursement rates outweigh other attributes, which was the main contribution of this study to this field. However, the proportion of other attributes in different disease scenarios still showed apparent changes, such as “waiting time” in the acute infectious diseases scenario and “hospital level” in the major disease scenario.

This study provides a theoretical reference for the refinement of many diseases' medical reimbursement policies, such as developing different reimbursement ratios for various common diseases and realizing rational configuration of medical resources. Respondents' strong preference for tertiary hospitals was shown in all scenarios, while their preference for care providers was not significantly affected. This indicates that, in order to implement hierarchical diagnosis and treatment, policymakers can pay more attention to the difference in medical insurance reimbursement among hospitals of different levels rather than simply strengthening the allocation of doctors in community hospitals. And this difference can not only be reflected in increasing the medical insurance reimbursement of community hospitals, but policymakers can also try to reduce the reimbursement ratio of tertiary hospitals flexibly.

Private hospitals also have great potential to reduce the medical burden on tertiary hospitals. Policymakers can consider providing policy inclinations for private hospitals with corresponding diagnosis and treatment qualifications. In addition to adjusting the reimbursement of medical insurance for hospitals of different levels, increasing the reimbursement types of medical insurance drugs may allow for more flexible adjustment of policies. For example, it can be stipulated that some commonly used medications for chronic non-communicable diseases can only be reimbursed in community hospitals. In three scenarios, different importance was attached to each attribute, which can be used for amending medical policies in the future, especially for hierarchical diagnosis and treatment. In the approaching studies, a detailed exploration of certain diseases and reimbursement rates can be conducted combined with hierarchical diagnosis and treatment background.

### Limitations

This study has some limitations. First, our study was conducted on the general population by hypothesis scenarios instead of real patients, so the results may not reflect their real choices. Second, the attributes in the questionnaire were determined by literature review combined with the actual situation. Although qualitative interviews and pilot tests have been carried out, these attributes may not fully represent the preferences of all Chinese residents, which is also one of the common disadvantages of DCE. Third, although qualitative interviews and pilot tests have been carried out, as this study applied a digital questionnaire, and due to the nature of the snowball sampling method, respondents with younger ages may account for a larger proportion, and this may result in an adverse effect on the representativeness of the data, further studies may be needed to investigate healthcare preference of people with older ages. Last, WTP is valued by model analysis with the attribute of “cost” as the reference, which plays a reference role as a whole, but does not mean that the respondents are willing to pay for a certain level.

## Conclusion

In conclusion, this study's results showed that reimbursement rates outweigh other attributes, which was the main contribution of this study to this field. However, the proportion of other attributes in different disease scenarios still showed apparent changes, such as “waiting time” in the acute infectious diseases scenario and “hospital level” in the major disease scenario. Moreover, this study provides a theoretical reference for the refinement of many diseases' medical reimbursement policies, such as developing different reimbursement ratios for various common diseases and realizing rational configuration of medical resources. In order to implement hierarchical diagnosis and treatment, policymakers can pay more attention to the difference in medical insurance reimbursement among hospitals of different levels rather than simply strengthening the allocation of doctors in community hospitals.

## Data availability statement

The original contributions presented in the study are included in the article/Supplementary material, further inquiries can be directed to the corresponding author/s.

## Ethics statement

The studies involving human participants were reviewed and approved by Medical Ethics Committee of Jinan University. The patients/participants provided their written informed consent to participate in this study.

## Author contributions

NY, TL, and W-KM: conceptualization and investigation. NY, TL, and YX: methodology, visualization, and writing—original draft preparation. LL and W-KM: supervision. NY, TL, XF, XM, MY, JD, ZT, E-wF, JH, BA, and CZ: writing—review and editing. All authors have read and agreed to the published version of the manuscript.

## Conflict of interest

The authors declare that the research was conducted in the absence of any commercial or financial relationships that could be construed as a potential conflict of interest.

## Publisher's note

All claims expressed in this article are solely those of the authors and do not necessarily represent those of their affiliated organizations, or those of the publisher, the editors and the reviewers. Any product that may be evaluated in this article, or claim that may be made by its manufacturer, is not guaranteed or endorsed by the publisher.
