# Supplementary material for: Healthcare preferences of the general Chinese population in the hierarchical medical system: A discrete choice experiment
Source: Front Public Health. 2022 Nov 17;10:1044550. doi: 10.3389/fpubh.2022.1044550 (PMC9713319; doi:10.3389/fpubh.2022.1044550)
Supplement: Supplementary file 1 [file Data_Sheet_1.docx]

**Appendix 1.**

The number of subgroups of each LC model of three types of diseases are decided based on the Akaike Information Criteria (AIC) and Bayesian Information Criteria (BIC).^31-34^ We create the BIC elbow plots according to Petras’ s study^34^, and find that the change from two to three groups was much smaller than the change from one to two groups, and among three types of disease scenarios, and from 3 groups, BIC become levelled off. Therefore, eventually, three subgroups are identified in the population of in each disease.

Figure A1. BIC of chronic non-infectious diseases (LC)


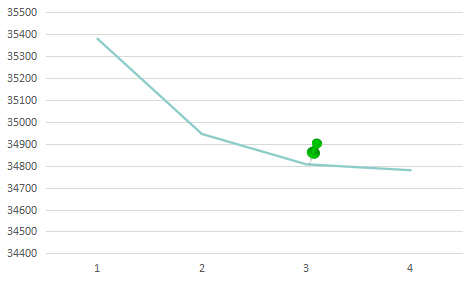


Figure A2. BIC of acute infectious diseases (LC)


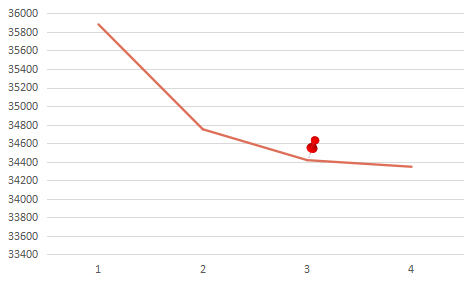


Figure A3. BIC of major diseases (LC)


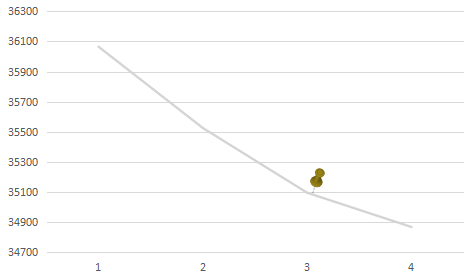


**Appendix 2.**

***Latent class Model (LC)***

***Acute infectious diseases***

According to the scenarios of acute infectious disease assumptions such as COVID-19 epidemic, we found some homogenous and heterogenous compared with the scenarios of chronic diseases. First of all, under the assumption of acute infectious disease, the attribute with the most importance was still the medical insurance reimbursement (35.94%). However, the heterogeneous rise up, the waiting time accounted was of much more attribute importance, 32.72% compared with 8.31% in the chronic disease scenarios. Additionally, the importance of hospital level shrink to 14.61% compared with 21.56% in the previous scenarios. Meanwhile, the importance of types of healthcare provider still remained the least importance with 2.42%.

The MNL indicated a monotonically decreasing preference from level Tertiary hospital to level private hospital (level Secondary hospital OR=0.083, 95% CI (0.857, 0.910), level Community hospital OR=0.701, 95% CI (0.679, 0.723) and level Private hospital OR=0.661, 95% CI (0.642, 0.682), p <0.001). Similarly, respondents’ preference of levels in other attributes also showed monotonically decreasing, except levels in the cost. And there is a monotonically increasing preference in medical insurance reimbursement, for example the level 100% (OR=2.763, 95% CI (2.652, 2.879).

The segment size of LCM in the acute infectious diseases is 69.46% (n=2668), 19.03% (n=731), 11.51% (n=442). Among the three segmented class, respondents in class 1 considers the reimbursement (51.01%) as the most essential attribute, while class 2 and 3 rank the waiting time as the most important in their trade-off.

***Major diseases***

The scenario of major diseases has some similarities with the scenario of chronic diseases. For instance, medical insurance reimbursement and the level of hospitals were ranked as the top two key attributes in respondents’ trade-off, accounted for 39.51% and 24.57% respectively. Especially the importance of hospital level, accounts more percentage importance than previous two scenarios. In addition, the care provider and cost still have the relatively low effect on respondents’ trade-off.

The MNL model in this scenario also highlighted the increasing preference along with the increasing level of hospital levels and medical reimbursement, and decreasing distance and cost.

The LCM indicate a segment size of 22.94% (n=881), 65.06% (n=2499), 12% (n= 461) with huge heterogeneous among these three classes. Specifically, respondents in class 1 believed reimbursement was the core factor, while class 2 and 3 attached the most importance on the level of hospital (39.46%) and waiting time (35.17%) respectively. Cost remained with the least importance in class 1 and 3, while types of care provider was the least preferred attribute in class 2.
